# Synthesis of Porous CoFe_2_O_4_ and Its Application as a Peroxidase Mimetic for Colorimetric Detection of H_2_O_2_ and Organic Pollutant Degradation

**DOI:** 10.3390/nano8070451

**Published:** 2018-06-21

**Authors:** Lihong Wu, Gengping Wan, Na Hu, Zhengyi He, Shaohua Shi, Yourui Suo, Kan Wang, Xuefei Xu, Yulin Tang, Guizhen Wang

**Affiliations:** 1 Key Laboratory of Advanced Materials of Tropical Island Resources (Hainan University), Ministry of Education, Haikou 570228, China; wulihongm@163.com (L.W.); wangengping001@163.com (Ge.W.); 17889886009@163.com (Z.H.); gjrshao726@163.com (S.S.); wangkanhd@163.com (K.W.); 18895627261@163.com (X.X.); yulintang321@163.com (Y.T.); 2 Key Laboratory of Tibetan Medicine Research, Northwest Institute of Plateau Biology, Chinese Academy of Sciences, Xining 810001, China; huna@nwipb.cas.cn (N.H.); yrsuo@nwipb.cas.cn (Y.S.)

**Keywords:** CoFe_2_O_4_, peroxidase-like activity, artificial enzyme mimetics, hydrogen peroxide

## Abstract

Porous CoFe_2_O_4_ was prepared via a simple and controllable method to develop a low-cost, high-efficiency, and good-stability nanozyme. The morphology and microstructure of the obtained CoFe_2_O_4_ was investigated by X-ray diffraction (XRD), X-ray photoelectron spectroscopy (XPS), transmission electron microscopy (TEM), high-resolution TEM (HRTEM), specific surface area and pore analysis, and Raman spectroscopy. The results show that the annealing temperature has an important effect on the crystallinity, grain size, and specific surface area of CoFe_2_O_4_. CoFe_2_O_4_ obtained at 300 °C (CF300) exhibits the largest surface area (up to 204.1 m^2^ g^−1^) and the smallest grain size. The peroxidase-like activity of CoFe_2_O_4_ was further verified based on the oxidation of peroxidase substrate 3,3’,5,5’-tetramethylbenzidine (TMB) in the presence of H_2_O_2_. The best peroxidase-like activity for CF300 should be ascribed to its largest surface area and smallest grain size. On this basis, an effective method of colorimetric detection H_2_O_2_ was established. In addition, the porous CoFe_2_O_4_ was also used for the catalytic oxidation of methylene blue (MB), indicating potential applications in pollutant removal and water treatment.

## 1. Introduction

Peroxidases are a kind of efficient redox enzymes that can catalyze the oxidation of enzyme substrate in the presence of H_2_O_2_. Owing to strong selectivity and high catalytic efficiency, they have been widely applied to various fields, such as biotechnology, chemical industry, biosensors, and immunoassays [1,2,3]. Unfortunately, there still exist some intrinsic drawbacks, such as being easy to denature in extreme environmental conditions, and the high cost of preparation and purification [4]. Therefore, the construction of novel and efficient artificial enzymes is very essential and has become an increasingly important focus. Over the past few decades, a vast variety of artificial enzymes have been explored to mimic the functions of natural enzyme, such as cyclodextrins, nanomaterials, DNA complexes, and Schiff base complex [5,6,7,8]. Among them, nanomaterials have attracted intensive attention because of their ease of preparation, low price, and good stability [9,10,11,12,13]. Additionally, the large specific surface and more activation centers of nanomaterials also greatly extend their application range [14].

To date, a large number of nanomaterials have been found to possess unexpected peroxidase-like activity (mimicking the functions of peroxidase), including Pt nanoclusters [15], AgVO_3_ nanobelts [16], MnO_2_ nanoparticles (NPs) [17,18,19], WO*_x_* quantum dots [20], Ag NPs [21], V_2_O_5_ nanowires [22], and Fe_3_O_4_ magnetic nanoparticles (MNPs) [23,24,25]. Additionally, a series of hybrid complexes based on metal nanomaterials have been investigated as peroxidase mimetics, such as GQDs/Ag NPs [26], Au nanocomposites [27,28], and FeSe-Pt@SiO_2_ nanospheres [29]. Furthermore, carbon-based nanomaterials, e.g., single-walled carbon nanotube [30], graphene oxide (GO) [6], C@Fe_3_O_4_ NPs [31], GQDs/CuO nanocomposites [32] were also explored to mimic the functions of naturally-occurring enzymes. In particular, magnetic nanomaterials have been widely applied in biology, medicine and environmental fields because of their low cost, better stability, ease of separation, and recyclability. For example, Wei and his co-workers reported that the Fe_3_O_4_ MNPs could be a highly-efficient peroxidase-like catalyst towards H_2_O_2_ reduction for biosensing applications [23]. Chen’s group found that magnetic carbon nitride nanocomposites possessed the superior peroxidase-like catalytic activity toward H_2_O_2_ and glucose and can be used as an enzyme-free biosensor [33]. Feng et al., reported the controllable synthesis of monodisperse CoFe_2_O_4_ nanoparticles by the phase transfer method and applied them in the degradation of methylene blue (MB) with H_2_O_2_ [34].

As a kind of high stability ferromagnetism nanomaterials, CoFe_2_O_4_ have been widely applied in lithium batteries, immunoassays, environmental science, and food chemistry [35,36,37]. Owing to the magnetic property for easy recyclability and high catalytic performance, CoFe_2_O_4_ is expected to be a good candidate for mimicking peroxidase [38,39]. In this study, we established a simple method to prepare porous CoFe_2_O_4_ with the controllable size of nanoparticles and specific surface area. The porous CoFe_2_O_4_ exhibited the excellent peroxidase-like catalytic activity and could be used to detect H_2_O_2_ and degrade MB. Benefiting from simple operation and high efficiency, CoFe_2_O_4_ materials have great potential applications in environmental protection and biotechnology.

## 2. Materials and Methods

### 2.1. Reagents

3,3′,5,5′-Tetramethylbenzidine (TMB) was purchased from Macklin (Shanghai, China). CoCl_2_·6H_2_O, FeSO_4_·7H_2_O, oxalic acid, methylene blue (MB), ethanol, H_2_O_2_ (30 wt %), isopropanol alcohol (IPA, a scavenger of hydroxyl radicals •OH) and other chemicals were all of analytical reagent grade and were obtained from Guangzhou Chemical Reagent factory (Guangzhou, China). All aqueous solutions were prepared with Milli-Q water (18.2 MΩ cm).

### 2.2. Preparation of CoFe_2_O_4_

CoFe_2_O_4_ samples were synthesized via a facile method. In a typical procedure, 2.5 g of CoCl_2_·6H_2_O and 5.6 g of FeSO_4_·7H_2_O were dissolved in 80 mL of deionized water and then transferred into oil bath heating at 80 °C, under vigorous stirring for 1 h. Subsequently, 30 mL 1 M oxalic acid solution was heated to boiling with magnetic stirring, and then added into the above solution slowly under constant stirring to form a final black precipitation, and then cooled by ice-water mixture. The black precipitates were collected by centrifugation and washed several times with water and ethanol, and further dried at 60 °C under vacuum for 12 h. Then, the precipitates were transferred to a tube furnace, heated to 300 °C, and maintained for 1 h. The temperature was raised at a heating rate of 1 °C min^−1^ [40]. The final product was denoted as CF300. Similarly, other CoFe_2_O_4_ samples prepared at 400 °C, 500 °C, 600 °C, and 700 °C were denoted as CF400, CF500, CF600, and CF700, respectively.

### 2.3. Characterization of CoFe_2_O_4_

The X-ray diffraction (XRD) patterns were collected on a Bruker D8 Advance X-ray diffractometer (Bruker Inc., Karlsruhe, Germany) with Cu Kα radiation (λ = 0.154056 nm). Scanning electron microscopy (SEM) images were observed by used a Hitachi S-4800N microscope (Hitachi Inc., Tokyo, Japan). The transmission electron microscopy (TEM) and high-resolution TEM (HRTEM) images were taken on a JEOL JEM-2100 microscope instrument (JEOL Inc., Tokyo, Japan) at an acceleration voltage of 200 kV. The chemical composition and elemental maps of the as-prepared samples were analyzed by energy-dispersive X-ray (EDX) spectroscopy using an EDX attachment to the SEM instrument. The X-ray photoelectron spectroscopy (XPS) data were acquired using AXIS SUPRA (Shimadzu Inc., Kyoto, Japan). The UV-vis absorbance spectra were recorded using a Lambda 750 s UV-vis-NIR absorption spectrophotometer (PerkinElmer Inc., Waltham, MA, USA). The hysteresis loop of the samples was obtained using the vibrating sample magnetometer (VSM, Lakeshore 7400, Westerville, OH, USA). The specific surface areas of the CoFe_2_O_4_ were recorded based on Brunauer-Emmett-Teller (BET) equation by an automatic nitrogen adsorption pore size distribution and specific surface analyzer (ASAP 2460, Atlanta, GA, USA). Raman spectroscopy was performed on a Renishaw inVia Reflex Raman microscope (Renishaw Inc., Gloucestershire, UK) using 532 nm green laser excitation.

### 2.4. Peroxidase-Like Activity Assay

To investigate the peroxidase-like activity of the prepared CoFe_2_O_4_ materials, a typical catalytic oxidation experiment was performed at room temperature with 100 μg/mL CF400 in 3 mL of phosphate-buffered saline (PBS, 20.0 mM, pH 3.5), using 0.8 mM TMB, and 4.0 mM H_2_O_2_ as the substrate. All the reactions were monitored in time scan mode at 652 nm, right after all of the reagents were added and mixed. Control groups included CF400 (100 μg/mL) with TMB (0.8 mM), CF400 (100 μg/mL) with H_2_O_2_ (4.0 mM), and TMB (0.8 mM) with H_2_O_2_ (4.0 mM) in 20.0 mM PBS (pH 3.0), respectively.

### 2.5. Optimal Conditions for H_2_O_2_ Detection

The effect of substrate H_2_O_2_ concentration (0–0.1 M), CF300 concentration (0–200 μg/mL), temperature (20–40 °C), and pH (2–8) on the peroxidase-like activity of CF300 were also investigated to ascertain the optimal conditions.

### 2.6. Analysis of Active Species

The active species generated in the reaction were detected by adding scavengers (IPA) into the reaction solutions [16]. The specific procedure was the same as the CF300 peroxidase-like activity assay experiments.

### 2.7. Steady-State Kinetic Study

A steady-state kinetic experiment was performed in a 3.0 mL reaction solution (20.0 mM PBS, pH = 3.0, 25 °C) with 20 μg/mL CF300. TMB and H_2_O_2_ were involved in the reaction as substrates. The steady-state kinetic data were collected by varying the concentration of one substrate while keeping the other substrate concentration constant, and the kinetic value was measured in time course mode at 652 nm [32,41]. The Michaelis-Menten constant was calculated by using the Lineweaver-Burk double reciprocal according to the equation: 1/*v* = (*K*_m_/*V*_max_) (1/[*S*]) + 1/*V*_max_ [42,43], where *v* is the initial velocity, *K*_m_ is the Michaelis constant, *V*_max_ is the maximal reaction velocity, and [*S*] represents the concentration of the substrate.

### 2.8. Methylene Blue Degradation

The catalytic activity of CoFe_2_O_4_ samples was demonstrated by degrading MB in aqueous solution. In a typical reaction, 0.015 mg of CF300 was added to 50 mL MB aqueous solution (10 mg/L), then 10 mL of aqueous H_2_O_2_ (30 wt %) was added in the reaction mixture. At various time intervals, a small quantity of the mixture solution was separated quickly by centrifugation. Finally, the supernatants were pipetted into a quartz cell and analyzed with UV-vis spectrophotometer to evaluate the catalytic degradation MB [41,44].

## 3. Results

### 3.1. Characterization of the CoFe_2_O_4_

**Figure 1:**
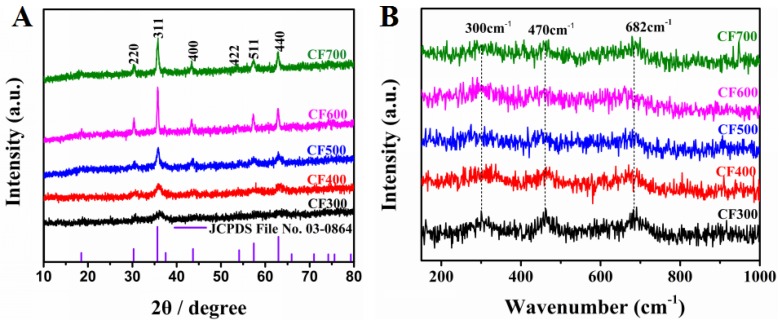
(**A**) XRD patterns and (**B**) Raman spectra of CoFe_2_O_4_ prepared at different temperatures.

The crystal structure and purity of the products were measured by XRD. Figure 1A displays XRD patterns of CoFe_2_O_4_ prepared at different temperatures. All characteristic peaks of samples match very well with the inverse spinel structure with the lattice parameters of a = 8.377 Å and c = 8.377 Å, which is consistent with the reported data (JCPDS file no. 03-0864). The synthesized temperature has a significant effect on the crystallinity of CoFe_2_O_4_. With the increase of the prepared temperature, the crystallization of samples increases obviously. Moreover, no other characteristic peaks of impurities are detected, indicating a pure phase of CoFe_2_O_4_ can be obtained using the present method. Figure 1B shows the Raman spectra of the as-synthesized samples. The low-frequency vibrations (below 600 cm^−1^) are belonged to the motion of oxygen around the octahedral lattice site whereas the higher frequencies can be attributed to oxygen around tetrahedral sites [45,46]. In this work, the mode at 682 cm^−1^ is a characteristic of the tetrahedral site. The bands at 470 cm^−1^ and 300 cm^−1^ correspond to Co^2+^ at octahedral sites [47].

**Figure 2:**
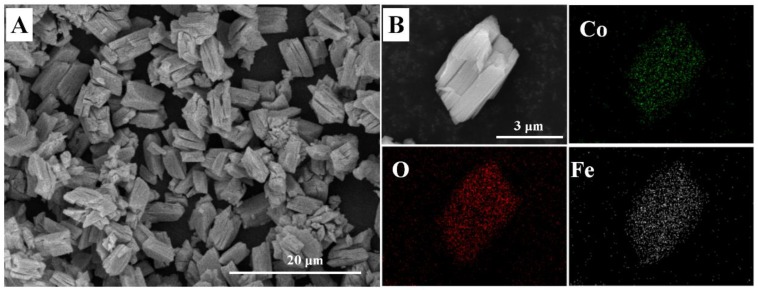
(**A**) SEM image of CF300 and (**B**) EDX elemental mappings.

The morphology of as-prepared CoFe_2_O_4_ is presented by SEM image. As shown in Figure 2A, CF300 show the rhombus-like polyhedron morphology with some fragments and cracks. They are several micrometers in size. Other CoFe_2_O_4_ samples obtained at different temperature also exhibit similar structure and morphology in Figure S1 (Supplementary Materials). The EDX elemental mappings (Figure 2B) and EDX spectrum (Figure S2) reveal the existence Co, O, and Fe elements. All three elements can maintain a uniform rhombus-like morphology, further demonstrating that Co, O, and Fe are homogeneously distributed in the sample.

**Figure 3:**
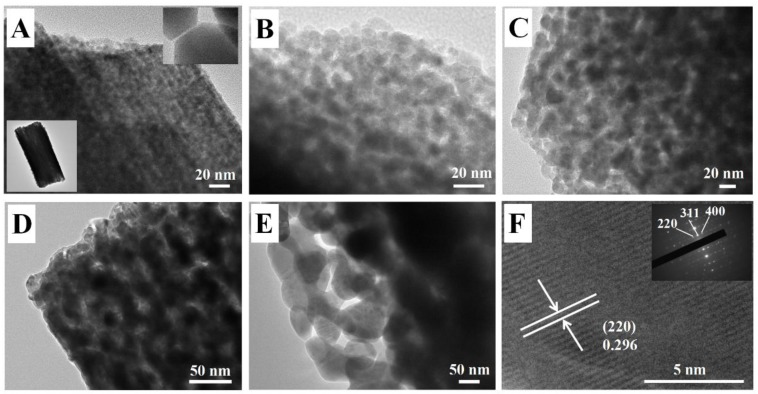
Typical images of (**A**) CF300; (**B**) CF400; (**C**) CF500; (**D**) CF600; and (**E**); CF700 (**F**); HRTEM image of CF300, and the inset in (**F**) is the SAED pattern.

The microstructure and morphology of the synthesized samples at different reaction temperature were further investigated by TEM. As observed from the TEM images in Figure 3A–E, all the samples exhibit a homogeneous mesoporous structure. When the synthesized temperature is increased from 300 °C to 700 °C, the pore size gradually enlarged and the pore density also decreased owing to the further crystallization and crystal growth of CoFe_2_O_4_ particles. In this work, the pores are located among the particles and mainly derived from a large amount of gases slowly release from oxalate particles during thermal decompositions [48]. Figure 3F displays the HRTEM image of CF300. The image highlights the crystalline lattice fringes with a distance of about 0.296 nm, which can be ascribed to the (220) plane of CoFe_2_O_4_. The inset in Figure 3F is the corresponding selected area electron diffraction (SAED) pattern, confirming that the sample has a monocrystalline structure. The diffraction spots can be indexed as inverse spinel-structured CoFe_2_O_4_, which is in accordance with the XRD result.

**Figure 4:**
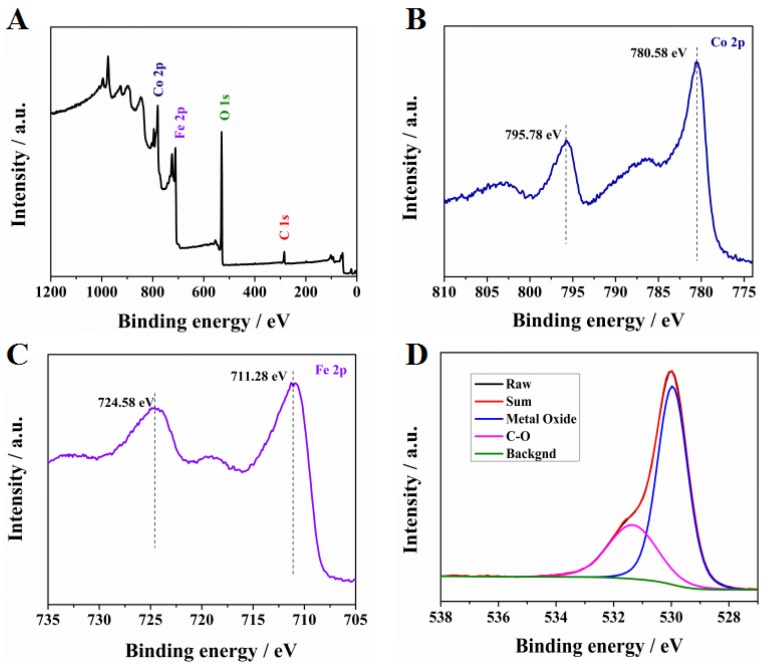
XPS spectra of CF300: (**A**) survey spectrum, high resolution XPS spectra of (**B**) Co 2p, (**C**) Fe 2p, and (**D**) O 1s.

The chemical composition and surface electronic state of the as-prepared CoFe_2_O_4_ samples were further studied by XPS. It can be seen in the XPS survey spectrum (Figure 4A) that the sample mainly contains three elements: Co, Fe, and O. The C 1s peak at around 284.8 eV could correspond to the signal from carbon contained in the instrument as calibration. In the Co 2p spectrum (Figure 4B), the peak located at about 780.58 eV corresponds to Co 2p_3/2_ with a satellite peak at 786.58 eV and the peak at 795.78 eV is ascribed to the Co 2p_1/2_. Figure 4C presents the Fe 2p orbital displaying the splitting peaks at binding energies of around 724.58 eV and 711.28 eV, which can be assigned to the Fe 2p_1/2_ orbital and Fe 2p_3/2_ orbital, respectively. These results are well in agreement with many other reports [49,50]. Figure 4D shows the high-resolution XPS spectra of the O 1s. Based on the previous reports, the signal peak located at 530.94 eV could be assigned to typical lattice oxygen in CoFe_2_O_4_. The peak located at 531.4 eV is assigned to C–O bonds. The XPS spectrum is in good agreement with the literature [51].

**Figure 5:**
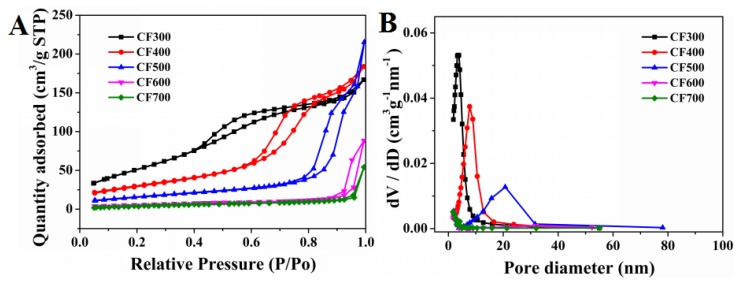
(**A**) N_2_ adsorption/desorption isotherms and (**B**) pore size distribution of the porous CoFe_2_O_4_.

N_2_ adsorption/desorption isotherms of the samples are depicted in Figure 5A. Typical type IV adsorption isotherms with an H3-type hysteresis loop are observed for all samples except CF700, indicating the mesoporous feature of the CoFe_2_O_4_ nanostructures. Figure 5B shows the relevant Barrett-Joyner-Halenda (BJH) pore size distribution of the porous CoFe_2_O_4_, in which there exists a sharp maximum at 3.5–20.6 nm, further indicating the material has a mesoporous structure. The porous trait will greatly enhance the surface area and active sites, and thus improve the catalytic performance [52]. The specific surface, pore diameter, and pore volume of these samples are shown in Table 1. We can see that, as the preparing temperature increases, the obvious decrease is found for the pore volume (0.228 to 0.019 cm^3^/g) and specific surface (204 to 18 m^2^/g). The magnetism of CF300 sample was determined by the hysteresis loop on VSM. It can be seen in Figure S3 that CF300 displays typical ferromagnetism at room temperature. The coercivity (Hc), saturation magnetization (Ms) and residual magnetization (Mr) are about 12 Oe, 8.372 emu/g and 0.064 emu/g respectively.

### 3.2. Peroxidase-Like Activity of CoFe_2_O_4_

**Figure 6:**
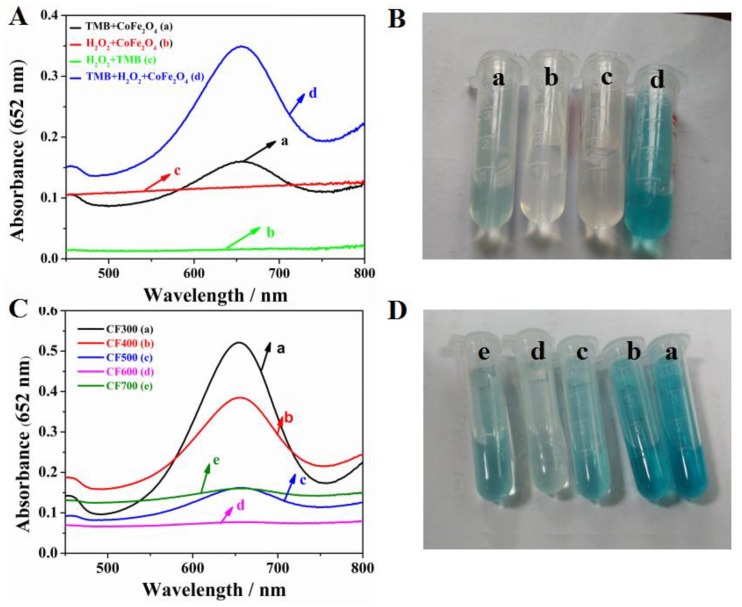
(**A**) UV-vis absorption spectra and (**B**) color changes in different reaction systems (a. TMB + CF400; b. H_2_O_2_ + CF400; c. H_2_O_2_ + TMB; d. TMB + H_2_O_2_ + CF400); (**C**) UV-vis absorption spectra and (**D**) color changes in the presence of different CoFe_2_O_4_ samples (a. CF300; b. CF400; c. CF500; d. CF600; e. CF700).

**Scheme 1:**
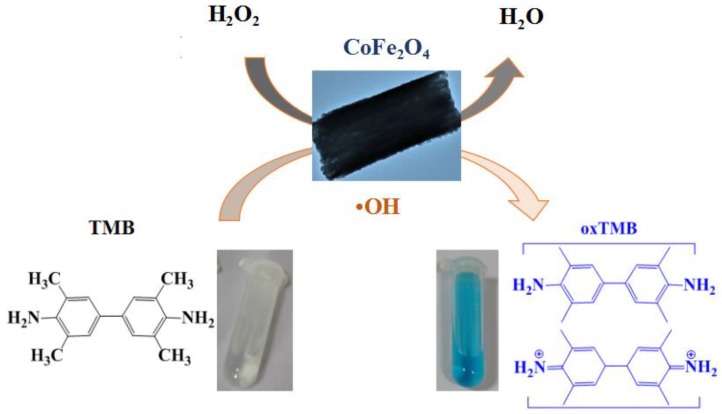
Schematic illustration of peroxidase-like activity for CF300.

To examine the peroxidase-like activity of the synthesized CoFe_2_O_4_, the catalytic oxidation of the typical peroxidase substrate TMB in the presence or absence of H_2_O_2_ was carried out (Figure 6A). Color changes of different systems can be observed from Figure 6B. The H_2_O_2_ + CF400 system and the H_2_O_2_ + TMB system show no absorption, while the TMB + H_2_O_2_ + CF400 system shows an obvious absorption peak at 652 nm, which indicates CF400 could act as a peroxidase to oxidize TMB to produce a blue color in the presence of H_2_O_2_. Interesting, the TMB + CF400 system shows a weak absorption, indicating that CF400 could catalyze the oxidation of TMB in the absence of H_2_O_2_ to produce a blue dye. The oxidase-like activity for TMB oxidation may originate from their catalytic ability to reduce dissolved oxygen [4]. Additionally, the peroxidase mimetic catalytic activities of CoFe_2_O_4_ at the different synthesized temperatures were tested and compared through the catalytic oxidation of TMB in the presence of H_2_O_2_. It can be seen from Figure 6C that CF300 exhibits the best peroxidase mimetic catalytic activity among the synthesized CoFe_2_O_4_ samples. The color changes of TMB with different CoFe_2_O_4_ samples are shown in Figure 6D. It can be found that all the CoFe_2_O_4_ samples prepared under different temperature possesses distinct peroxidase mimetic catalytic activities. CF300 system shows the most obvious blue color in comparison with that of CF400, CF500, CF600, and CF700. The most superior peroxidase mimetic activity of CF300 could be mainly owing to the rich catalytic activity sites provided by the largest specific surface area. As shown in Scheme 1, H_2_O_2_ reduction first takes place in the presence of chromogenic electron donor TMB, and then accelerates by partial electron transferring to the surface of CF300. Subsequently, the TMB is oxidized through one electron transfer and the color of the solution changes to blue.

### 3.3. Optimal Condition for H_2_O_2_ Detection

**Figure 7:**
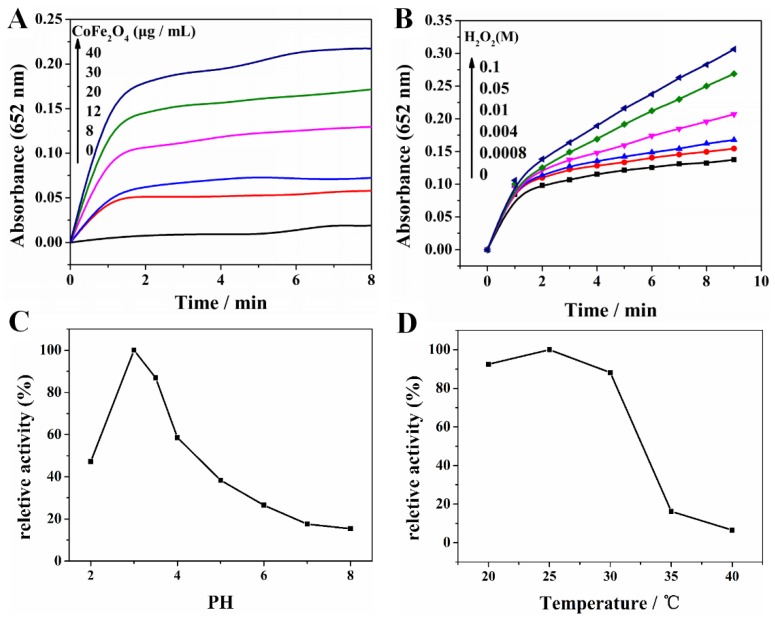
(**A**) Time-dependence absorbance at 652 nm of the 0.08 mM TMB reaction solution in the absence or presence of different doses of CF300 in 20 mM PBS (pH = 3.0) with 0.8 mM H_2_O_2_ at room temperature. (**B**) Time-dependence absorbance at 652 nm of the 0.4 mM TMB reaction solution in the absence or presence of different concentrations of H_2_O_2_ in 20 mM PBS (pH = 3.0) with 20 μg/mL CF300 at room temperature. Dependence of peroxidase-like activity of CF300 on pH (**C**) and temperature (**D**).

In order to investigate the intrinsic catalytic activities of CF300, further experiments were carried out with various CF300 and H_2_O_2_ concentrations. Figure 7A shows that the peroxidase-like catalytic reaction rate increases with the elevating concentrations of CF300. For the convenience of operation, the concentration of CF300, 20 μg/mL, is used in the following experiments. Previous studies showed that the catalytic activities of horseradish peroxidase (HRP) could be inhibited at excess concentrations of H_2_O_2_ [53]. Figure 7B depicts the time course-dependent absorbance change at 652 nm for different concentrations of H_2_O_2_. The catalytic reaction rates increase with the increase of H_2_O_2_ concentration, and no inhibition is found in the catalytic reaction at high H_2_O_2_ concentrations, which suggests a more stable catalytic activity for CF300 than that of HRP at high H_2_O_2_ concentration. The 4.0 mM is chosen as the optimal H_2_O_2_ concentration in the following experiments.

Similar to HRP and other mimetic enzymes, the catalytic relative activity of CF300 is dependent on the pH and temperature. Hence, we investigated the peroxidase-like activity of CF300 through varying the pH from 2 to 8 and the temperature from 20 °C to 40 °C. As shown in Figure 7C, the peroxidase-like activity of CF300 is much higher in weakly acidic solutions than in alkaline and neutral solutions. The maximum absorption for CF300 catalytic system appears at pH 3.0. In addition, the catalytic activity of CF300 is significantly affected by the temperature (Figure 7D). The catalytic activity offers an upgrade, firstly, with the increasing temperature. Subsequently, it starts to decline when the temperature is higher than 25 °C. Thus, for the convenience of operation, the 25 °C is selected as the experimental temperature.

### 3.4. Catalytic Mechanism Study

In the presence of H_2_O_2_, CF300 could catalyze H_2_O_2_ to form the oxidized intermediate •OH radicals and subsequently oxidize TMB to produce blue dye. In order to confirm the catalytic mechanism, the •OH radical scavenger IPA was used in the CF300-TMB-H_2_O_2_ system, where IPA would easily react with •OH, leading to the decrease of the absorption at 652 nm and color fading of the system [2]. The corresponding chemical equation was:

CH_3_CH(OH)CH_3_ + •OH → CH_3_CH(O)CH_3_ + H_2_O

**Figure 8:**
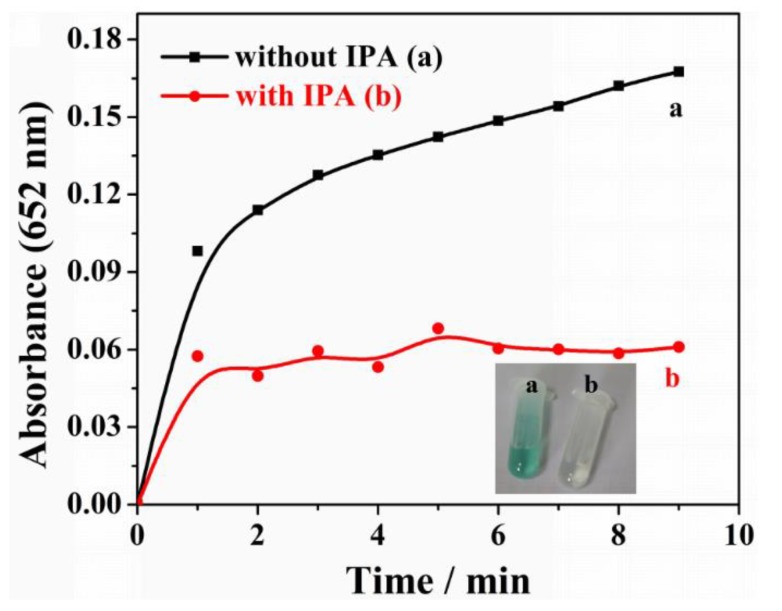
Time-dependent absorbance at 652 nm of the 0.4 mM TMB reaction solutions in the absence or presence of scavengers (IPA) in 20 mM PBS (pH = 3) with 20 μg/mL CF300 and 0.8 mM H_2_O_2_.

Figure 8 shows an apparent color fading and absorption decrease at 652 nm for the system with IPA, confirming that CF300 could catalytically activate H_2_O_2_ to generate •OH radicals, and then oxidize TMB to produce a TMB oxide with blue color. Obviously, these results indicate that •OH radicals play a key role in the catalytic reaction.

### 3.5. Steady-State Kinetic Assay

**Figure 9:**
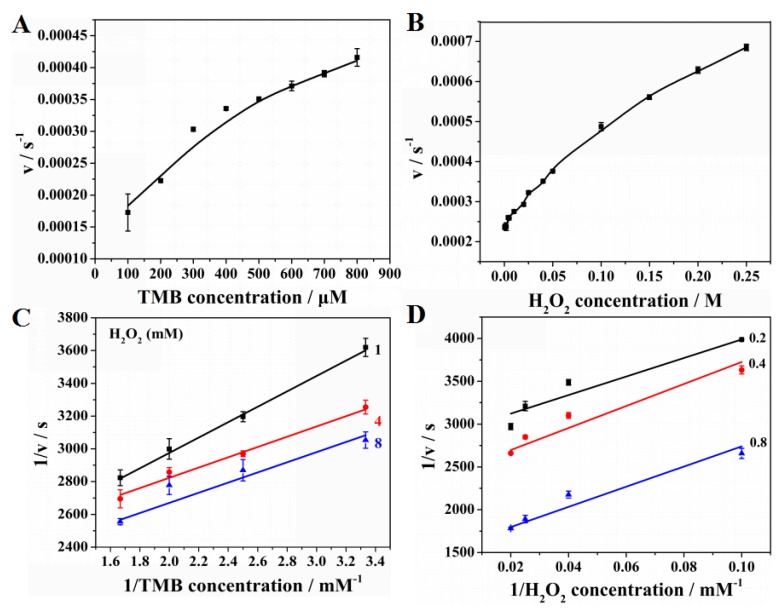
Steady-state kinetic assays of CF300, the reaction velocity (*v*) was measured using 20 μg/mL of CF300 in 20 mM PBS (pH = 3) at room temperature. The TMB concentration was varied and the concentration of H_2_O_2_ was 4 mM (**A**); the H_2_O_2_ concentration was varied and the concentration of TMB was 0.4 mM (**B**); and the double-reciprocal plots of peroxidase-like activity of CF300 with a fixed concentration of one substrate relative to varying concentration of the other substrate (**C**,**D**).

The catalytic steady-state kinetic of CF300 was evaluated by using TMB and H_2_O_2_ as substrates (Figure 9). The kinetic experiments were performed by only varying the concentration of TMB or H_2_O_2_ while keeping the other substrate concentration constant. As illustrated in Figure 9A,B, the Michaelis–Menten curves were obtained for both TMB and H_2_O_2_ in a certain concentration range. Accordingly, a series of initial reaction rates were obtained, and the double reciprocal plots (the reciprocal initial velocity vs. the reciprocal substrate concentration) were drawn as demonstrated in Figure 9C,D. The Michaelis–Menten constant (*K_m_*) and maximal reaction velocity (*V*_max_) for this system were calculated using Lineweaver–Burk plots and the Michaelis-Menten equation. The *K_m_* (TMB) of the CF300 is 0.387 mM, which is lower than the reported value of HRP (0.434 mM), suggesting that CF300 has higher affinity for TMB than HRP (Table 2). On the other hand, the *K_m_* (H_2_O_2_) of the CF300 is 8.89 mM, significantly higher than that of 3.7 mM for HRP [25], indicating that CF300 has lower affinity for H_2_O_2_. Additionally, the peroxidase-like catalytic reaction based on CF300 followed the typical Michaelis-Menten behavior [2,13] towards both substrates: TMB and H_2_O_2_. Additionally, the double-reciprocal plots (Figure 9C,D) exhibited the characteristic parallel lines of a ping-pong mechanism, indicating that CF300 bound and reacted with the first substrate and then released the first product before reacting with the second substrate, which is similar to that of HRP [2,25].

### 3.6. Detection of H_2_O_2_ and Oxidative Degradation of Methylene Blue

**Figure 10:**
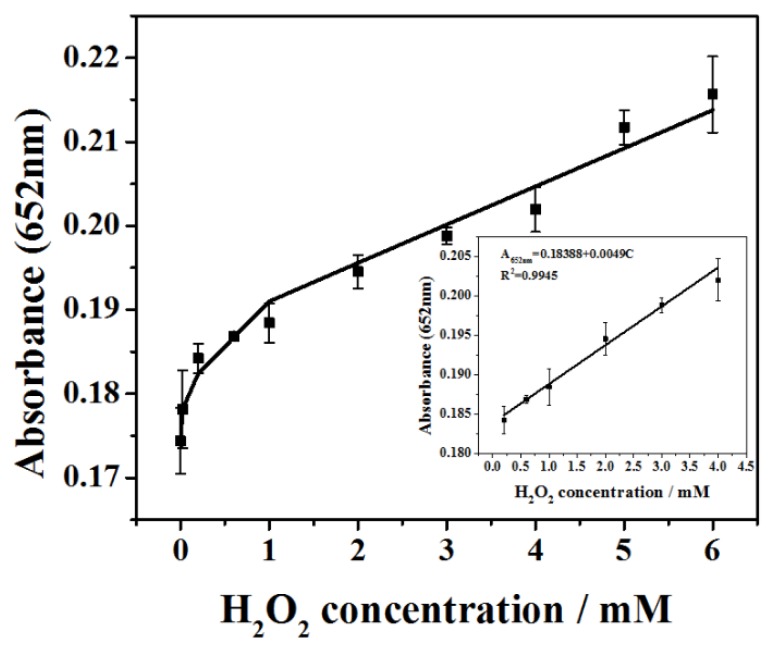
A concentration response curve H_2_O_2_ detection. Inset: the linear calibration plot.

On the basis that peroxidase mimics the activity of the CoFe_2_O_4_ materials, we developed a facile and label-free colorimetric approach to detect H_2_O_2_. As shown in Figure 10, a typical H_2_O_2_ concentration response curve under the optical conditions is measured. The detection range for H_2_O_2_ is from 0.02 to 6 mM. The linear regression equation is *A*_652nm_ = 0.18388 + 0.0049C (mM), with a correlation coefficient of 0.9945. The detection limit for H_2_O_2_ is 0.02 mM, suggesting that the optical biosensing system built in this study can be applicable to the H_2_O_2_ determination.

**Figure 11:**
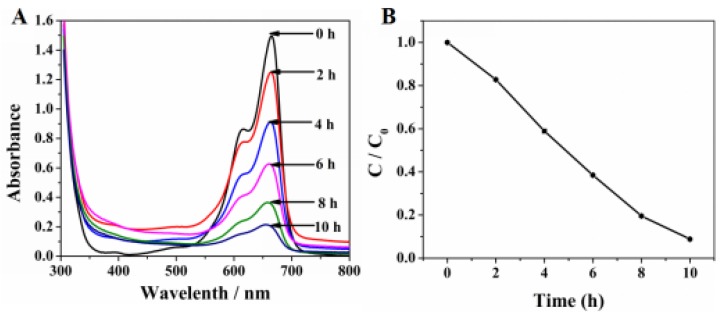
(**A**) UV-vis spectra obtained during the MB oxidative degradation over CF300; and (**B**) the MB degradation rate at various reaction time.

Some studies confirmed that nanozymes could be used to remove pollutants from waste water [54,55,56,57]. Based on the excellent catalytic activity of CF300, we further investigated its MB degradation ability. Similar to other enzyme mimic reactions, the maximum UV-vis absorption (*A*_max_) of MB aqueous solution is located at 665 nm [44]. Figure 11A shows the UV-vis absorption spectra of MB aqueous solution in the presence of CF300 and H_2_O_2_ at different reaction time. A sharp decrease for the absorption intensity of MB is observed with the increasing reaction time, the band at 665 nm become very weak and broad, and the removal rate of MB can reach 91.2% at 10 h (Figure 11B), suggesting that the MB has nearly completed degradation.

## 4. Conclusions

In summary, we developed a simple and controllable method for porous CoFe_2_O_4_ preparation. The specific surface area of CoFe_2_O_4_ materials was dependent on the annealing temperature and the CF300 exhibited a much higher specific surface area (204.1 m^2^ g^−1^) than other CoFe_2_O_4_ samples. The large specific surface area of CF300 endowed them more actives which resulted in the enhanced intrinsic peroxidase mimics activity. Additionally, based on the peculiarity, CF300 had excellent catalytic activity for the degradation of MB. Owing to the facile preparation, low cost, large specific surface area, and magnetic properties, the present CoFe_2_O_4_ showed great potential for applications in environmental protection, biomedical analysis, and other related areas.

## Figures and Tables

**Table 1:** Specific surface, pore diameter, and pore volume of synthesized CoFe_2_O_4_ samples.

Sample	Specific Surface (m^2^/g)	Pore Diameter (nm)	Pore Volume (cm^3^/g)
CF300	204.10	4.47	0.228
CF400	112.17	8.84	0.248
CF500	58.44	14.29	0.209
CF600	21.78	5.56	0.030
CF700	18.25	4.18	0.019

**Table 2:** Comparison of *K_m_* and *V*_max_ between CF300 and HRP for H_2_O_2_ and TMB.

Catalysts	Substances	*K_m_* (mM)	*V*_max_ (M/s)	References
CoFe_2_O_4_	H_2_O_2_	8.89	1.93 × 10^−8^	This work
CoFe_2_O_4_	TMB	0.387	2.90 × 10^−8^	This work
HRP	H_2_O_2_	3.7	8.71 × 10^−8^	[10]
HRP	TMB	0.434	10.00 × 10^−8^	[10]

## Supplementary Materials

The following are available online at http://www.mdpi.com/2079-4991/8/7/451/s1.
