# Supplementary material for: Synthesis of Porous CoFe2O4 and Its Application as a Peroxidase Mimetic for Colorimetric Detection of H2O2 and Organic Pollutant Degradation
Source: Nanomaterials (Basel). 2018 Jun 21;8(7):451. doi: 10.3390/nano8070451 (PMC6071025; doi:10.3390/nano8070451)
Supplement: Supplementary file 1 [file nanomaterials-08-00451-s001.pdf]

# Supplementary Material

# Synthesis of porous $CoFe_2O_4$ and its application as peroxidase mimetics for colorimetric detection of $H_2O_2$ and organic pollutants degradation

**Lihong Wu[1], Gengping Wan[1], Na Hu[2], Zhengyi He[1], Shaohua Shi[1], Yourui Suo[2], Kan Wang[1], Xuefei Xu[1], Yulin Tang[1] and Guizhen Wang[1,*]**

[1] Key Laboratory of Advanced Materials of Tropical Island Resources (Hainan University), Ministry of Education, Haikou 570228, China

[2] Key Laboratory of Tibetan Medicine Research, Northwest Institute of Plateau Biology, Chinese Academy of Sciences, Xining 810001, China

[*] Correspondence: wangguizhen@hainu.edu.cn; wangguizhen0@hotmail.com; Tel.: +86-0898-66268172.

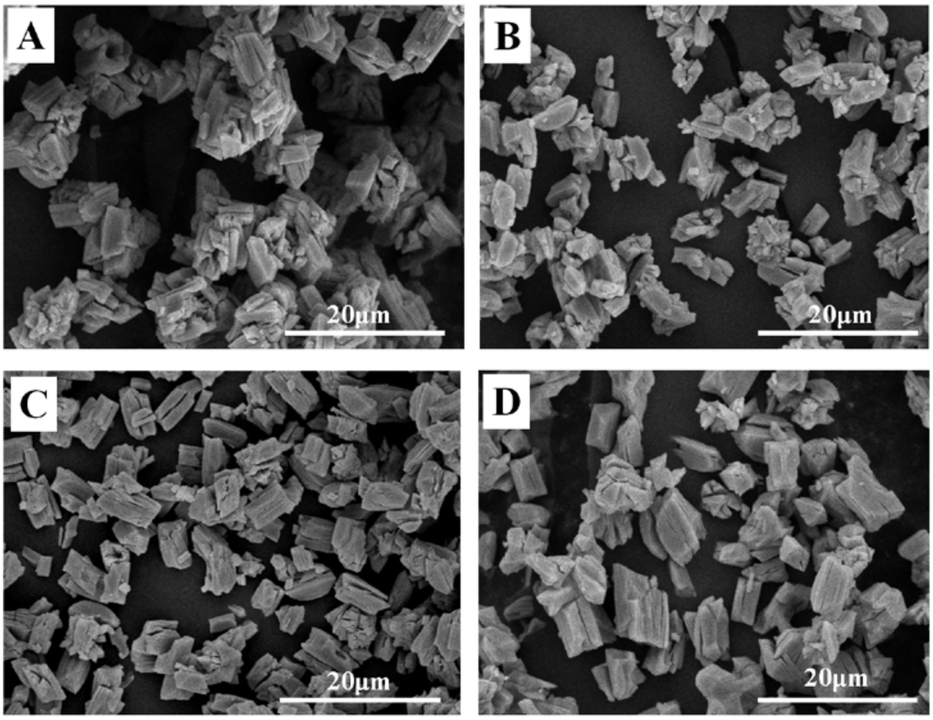


**Figure S1.** SEM images of CF400 (A), CF500 (B), CF600 (C) and CF700 (D).

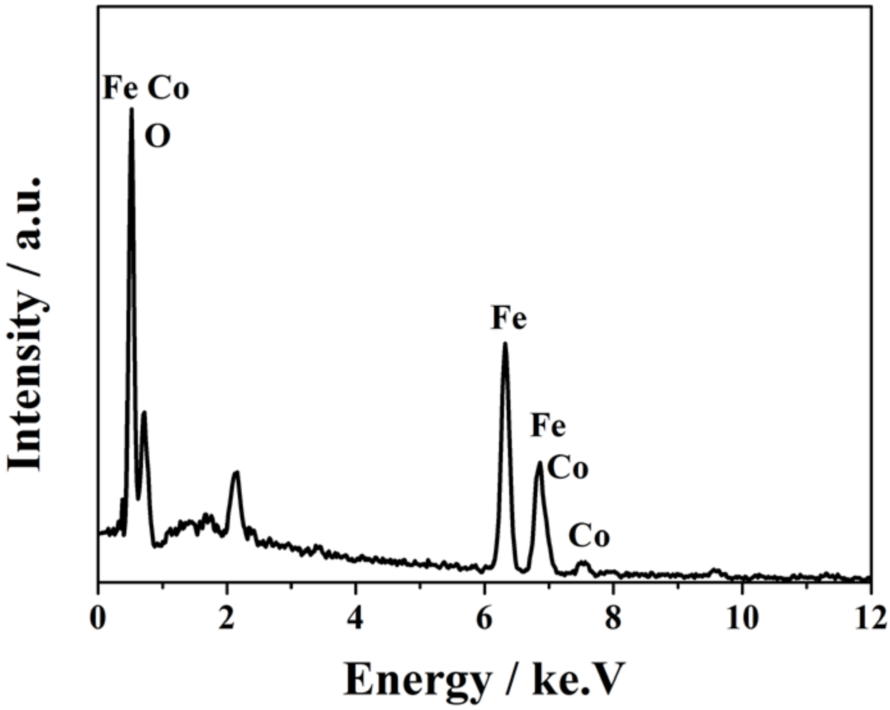


**Figure S2.** The EDX spectrum of CF300.

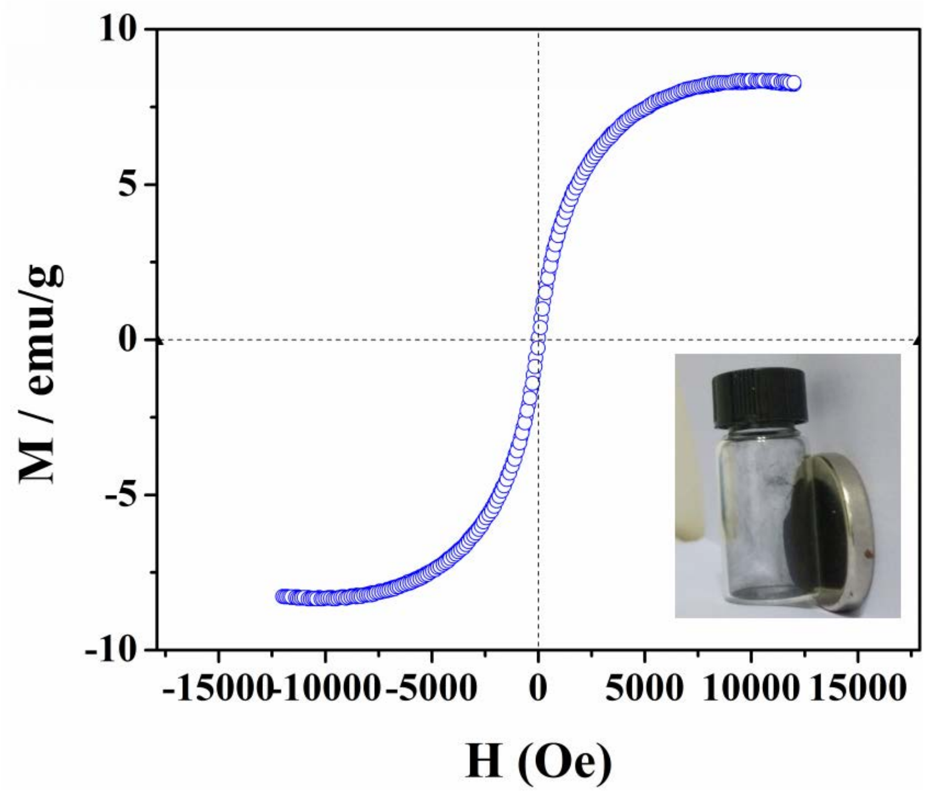


**Figure S3.** The magnetic hysteresis loop.
